# A re-consideration of the taxonomic status of *Nebria lacustris* Casey (Coleoptera, Carabidae, Nebriini) based on multiple datasets – a single species or a species complex?

**DOI:** 10.3897/zookeys.147.2082

**Published:** 2011-11-16

**Authors:** David H. Kavanaugh, Sophie L. Archambeault, Peter D. Roopnarine, Joel Ledford

**Affiliations:** 1 Department of Entomology, California Academy of Sciences, San Francisco, California 94118; 2 Department of Invertebrate Zoology and Geology, California Academy of Sciences, San Francisco, California 94118

**Keywords:** Coleoptera, Carabidae, Nebriini, *Nebria*, *Boreonebria*, Appalachian Mountains, phylogeny, endemism, vicariance, French Broad River, “Driftless Zone”

## Abstract

This study gathered evidence from principal component analysis (PCA) of morphometric data and molecular analyses of nucleotide sequence data for four nuclear genes (28S, TpI, CAD1, and Wg) and two mitochondrial genes (COI and 16S), using parsimony, maximum likelihood, and Bayesian methods. This evidence was combined with morphological and chorological data to re-evaluate the taxonomic status of *Nebria lacustris* Casey sensu lato. PCA demonstrated that both body size and one conspicuous aspect of pronotal shape vary simultaneously with elevation, latitude, and longitude and served to distinguish populations from the southern Appalachian highlands, south of the French Broad, from all other populations. Molecular analyses revealed surprisingly low overall genetic diversity within *Nebria lacustris* sensu lato, with only 0.39% of 4605 bp varied in the concatenated dataset. Evaluation of patterns observed in morphological and genetic variation and distribution led to the following taxonomic conclusions: (1) *Nebria lacustris* Casey and *Nebria bellorum* Kavanaugh should be considered distinct species, which is a NEW STATUS for *Nebria bellorum*. (2) No other distinct taxonomic subunits could be distinguished with the evidence at hand, but samples from northeastern Iowa, in part of the region known as the “Driftless Zone”, have unique genetic markers for two genes that hint at descent from a local population surviving at least the last glacial advance. (3) No morphometric or molecular evidence supports taxonomic distinction between lowland populations on the shores of Lake Champlain and upland populations in the adjacent Green Mountains of Vermont, despite evident size and pronotal shape differences between many of their members.

## Introduction

Thomas Lincoln Casey described *Nebria lacustis* in 1913, based on specimens from Wisconsin (type locality = Bayfield, Bayfield County) and Minnesota. In the same paper (Casey 1913: 56), he described *Nebria expansa*, based on two female specimens, one from “Texas” and the other from Indiana. He included these two species, along with *Nebria pallipes* Say (1823: 78), in his “Group *pallipes*”. He distinguished *Nebria lacustris* from *Nebria expansa* on the basis of overall size (*Nebria lacustris* adults being smaller than those of *Nebria expansa*), relative size of the pronotum (which he described as larger and wider, especially anteriorly, in *Nebria expansa* adults than in *Nebria lacustris*), and distinctness of the punctures of the elytral striae (which he described as more deeply and distinctly punctate in *Nebria lacustris* than *Nebria expansa*).

Both Leng (1920) and Csiki (1927) listed *Nebria lacustris* and *Nebria expansa* as distinct species in their catalogs. However, Bänninger (1925) listed *Nebria lacustris* in his treatment of the Nebriini but did not mention *Nebria expansa*. Bell (1955) suggested that Bänninger’s omission indicated that “he considered *expansa* to be a synonym” of *Nebria lacustris* and added that, based on his review of “the materials in the United States National Museum, including the types of both species, I believe that *expansa* is at most a poorly defined geographic race of *lacustris*.” He further suggested that “typical *lacustris* is from more northern localities, but typical *expansa* and intermediates are represented from both southern and northern localities.

**Figure 1.**
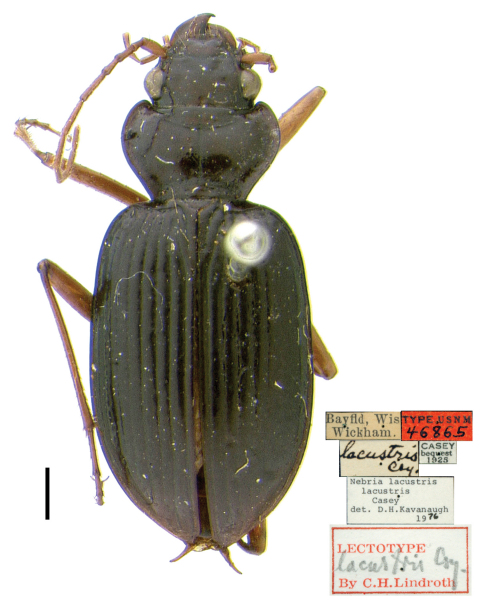
*Nebria lacustris* Casey, lectotype female, dorsal habitus and labels; scale line = 1.0 mm

**Figure 2.**
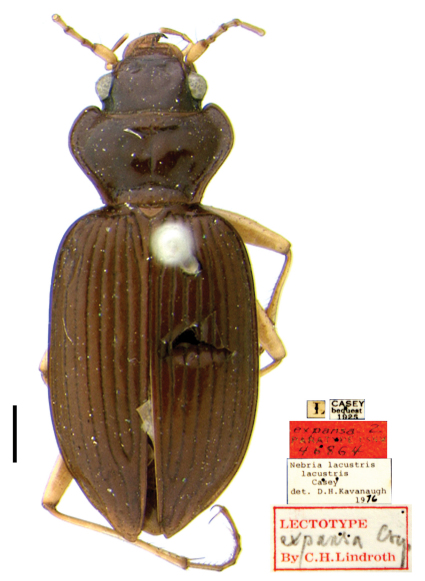
*Nebria expansa* Casey, lectotype female, dorsal habitus and labels; scale line = 1.0 mm

Lindroth (1961) was the first to formally treat *Nebria expansa* as a synonym of *Nebria lacustris*. In a subsequent paper (Lindroth 1975), he designated lectotypes for *Nebria lacustris* (p. 112) (Fig. 1) and *Nebria expansa* (p. 112, type area = “Texas”; amended in an addendum (p. 147) to Indiana, based on a recommendation from Kavanaugh (in litt.)) (Fig. 2). He concluded his treatment of *Nebria expansa* in the latter paper (p. 112), by adding the following: “Regarded as a synonym of *lacustris* Csy. (Lth. 1961: 77) but possibly at least subspecifically distinct (Bell in litt.).”

**Table 1.** Taxon samples and localities

Taxon	Extraction Code #	Locality
*Nebria (s. str.) brevicollis* Fabricius	DHK0717	U.S.A., Oregon, Polk County, 1.5 miles W of Dallas
*Nebria (Boreonebria) gyllenhali* (Schönherr)	DHK0010	RUSSIA, Buryat Republic, Khamar-Daban Mountains, Tankhoy
*Nebria (Boreonebria) crassicornis* Van Dyke	DHK0021	U.S.A., Washington, Olympic National Park, Hurricane Ridge
*Nebria (Boreonebria) subdilatata* Motschulsky	DHK0012	RUSSIA, Buryat Republic, Khamar-Daban Mountains, Tankhoy
*Nebria (Boreonebria) baicalica* Motschulsky	DHK0386a	RUSSIA, Irkutsk Region, Lake Baikal at Bolschie Koty
*Nebria (Boreonebria) nivalis* (Paykull)	DHK0387a	CANADA, Nunavut, Baffin Island, Glasgow Inlet, Kimmirut
*Nebria (Boreonebria) nivalis* (Paykull)	DHK0388a	U.S.A., Alaska, Katmai National Park, Brooks Lake
*Nebria (Boreonebria) gouleti* Kavanaugh	DHK0006	U.S.A., Idaho, Idaho County, Selway River, 7.5 miles SE of Lowell
*Nebria (Boreonebria) gouleti* Kavanaugh	DHK0027	U.S.A., Idaho, Idaho County, Salmon River at Riggins
*Nebria (Boreonebria) hudsonica* LeConte	DHK0381a	U.S.A., Wyoming, Sublette County, Hoback River
*Nebria (Boreonebria) lacustris* Casey	DHK0066	U.S.A., Iowa, Hardin County, Iowa River at Steamboat Rock
*Nebria (Boreonebria) lacustris* Casey	DHK0067	U.S.A., Iowa, Hardin County, Iowa River at Steamboat Rock
*Nebria (Boreonebria) lacustris* Casey	DHK1196	U.S.A., Vermont, Washington County, Ridley Creek
*Nebria (Boreonebria) lacustris* Casey	DHK1197	U.S.A., Vermont, Washington County, Ridley Creek
*Nebria (Boreonebria) lacustris* Casey	DHK1198	U.S.A., Vermont, Washington County, Ridley Creek
*Nebria (Boreonebria) lacustris* Casey	DHK1203	U.S.A., Vermont, Chittenden County, Burlington, Lake Champlain, Oakledge Park
*Nebria (Boreonebria) lacustris* Casey	DHK0004	U,S.A., Maryland, Montgomery County, Potomac River at Plummers Island
*Nebria (Boreonebria) lacustris* Casey	DHK0383	U,S.A., Maryland, Montgomery County, Potomac River at Plummers Island
*Nebria (Boreonebria) lacustris* Casey	DHK0384	U,S.A., Maryland, Montgomery County, Potomac River at Plummers Island
*Nebria (Boreonebria) lacustris* Casey	DHK1381	U,S.A., Maryland, Montgomery County, Potomac River at Plummers Island
*Nebria (Boreonebria) lacustris* Casey	DHK1384	U,S.A., Maryland, Montgomery County, Potomac River at Plummers Island
*Nebria (Boreonebria) lacustris* Casey	DHK0068	U.S.A., North Carolina, Burke County, Linville River below Linville Falls
*Nebria (Boreonebria) bellorum* Kavanaugh	DHK0509	U.S.A., Tennessee, Sevier County, Middle Fork of the Little Pigeon River
*Nebria (Boreonebria) bellorum* Kavanaugh	DHK0510	U.S.A., Tennessee, Sevier County, Middle Fork of the Little Pigeon River
*Nebria (Boreonebria) bellorum* Kavanaugh	DHK0511	U.S.A., Tennessee, Sevier County, Middle Fork of the Little Pigeon River

**Figure 3.**
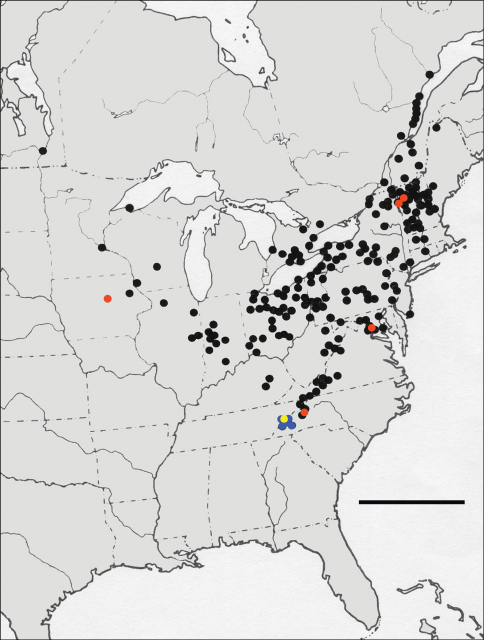
Map of known localities for members of the *lacustris* species subgroup of *Nebria*; black and red dots = *Nebria lacustris* LeConte; blue and yellow dots = *Nebria bellorum* Kavanaugh; red and yellow dots, respectively, denote localities from which DNA samples were obtained (see also Table 1); scale line = 500 km;

As part of his study of the *Nebria* of North America (Kavanaugh 1978), the lead author [DHK] had the opportunity to review all the material representing *Nebria lacustris* deposited in more than 100 institutional and private collections around the world and available by the early 1970’s – a total of nearly 1800 specimens. Distributional data associated with these specimens revealed a roughly V-shaped cumulative geographical distribution in eastern North America (Fig. 3), extending from the north shore of the Saint Lawrence estuary in southcentral Québec, south southwest to the southern Appalachian Mountains of western North Carolina and eastern Tennessee, and northwest to eastern Illinois, Iowa, and Minnesota, and southeastern Manitoba. No localities represented among the specimens examined were within 930 km (580 miles) of the state of Texas, which strongly suggested that Casey’s record of *Nebria expansa* for that state was based on either a mislabeling or misinterpretation of the label data. For example, there is a town named Texas in Dearborn County, Indiana, on the west bank of the Ohio River, which is within the range of this species, but from which no other known specimens have been collected. Lindroth’s (1975) amended type locality (see above) for *Nebria expansa* was based on this information.

**Figures 4–5.**
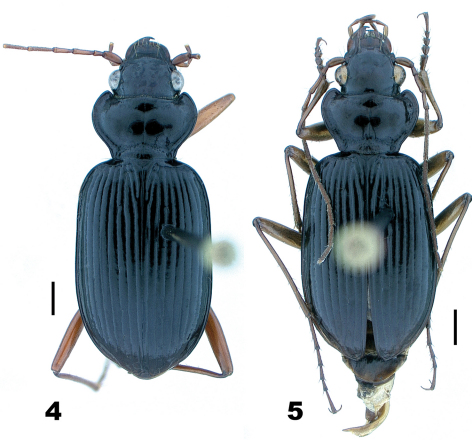
*Nebria lacustris* Casey, male, dorsal habitus; scale line = 1.0 mm **4** Fleury Bay, Lake Champlain, Grand Isle County, Vermont **5** Ridley Brook, Washington County, Vermont.

In May and June, 1973, DHK and Henri Goulet collected widely within the range of *Nebria lacustris*, from Tennessee and North Carolina north to southern Québec and west to Indiana, and gathered specimens and data on habitat distribution for this species across this region. They had the pleasure of visiting Ross and Joyce Bell at their home in Burlington, VT during this trip and collecting with them at several of Ross and Joyce’s favorite sites in that area. It was during this visit that the Bells shared their suspicion that there may be two species or subspecies, distinct in form and habitat preference, included under the current *Nebria lacustris* species concept: one (Fig. 4) confined, at least in the Burlington area, to the lowland shores of Lake Champlain, the other (Fig. 5) to cool shaded streams at higher elevations in the nearby Green Mountains. Specimens in their collection from these respective areas certainly appeared to differ in size and form, so DHK borrowed their material for further study.

**Figures 6–7.**
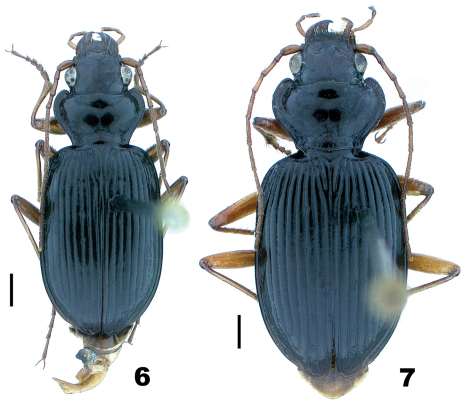
Dorsal habitus; scale line = 1.0 mm **6** *Nebria bellorum* Kavanaugh, male, Smokemont, Swain County, North Carolina **7** *Nebria lacustri*s Casey, female, Harpers Ferry, Jefferson County, West Virginia.

Detailed examination of all of the specimens of *Nebria lacustris* acquired through loans (including the Bells’ Vermont specimens) and fieldwork showed considerable variation in overall size, in relative size, proportions, and shape of the pronotum, and in relative position of the midlateral seta in relation to pronotal length. Some of the observed variation was clearly intrapopulational, but some also appeared to be correlated with latitude, longitude, and altitude, hence geography. In an attempt to clarify patterns of geographical variation, if any, in these features, DHK recorded several measurements (see Morphological methods below) for each of the available specimens; however, his morphometric analysis was limited to plotting various character bivariate scatterplots, none of which demonstrated a clear pattern of geographical variation. What was clear, however, was that specimens from the southern Appalachian mountains (Fig. 6) are most similar in size to those from the Green Mountains of Vermont, White Mountains of New Hampshire, and the north shore of the Saint Lawrence estuary in Québec, and much less similar in size and shape to specimens from more proximate, southerly locations, especially the nearest areas to the north and northeast (Fig. 7). The region of this phenotypic discontinuity corresponds with one of the best-known distributional barriers in unglaciated portions of eastern North America, namely the French Broad River. Barr (1969) noted that “the valley of the French Broad River forms the most striking distributional barrier in the high [Southern Appalachian] mountains, but its effect is variable depending on the species.” He recorded species or subspecies pairs vicariant across the French Broad River valley in several groups of carabids, including cychrines (*Scaphinotus (Steneridia*) spp. and ssp., *Sphaeroderus* ssp.) trechines (*Trechus* spp.), bembidiines (*Anillinus* spp.), and pterostichines (*Pterostichus (Gastrellarius)* spp.) (Barr 1969) and in pselaphine staphylinids (*Arianops* spp.) Barr (1974).

**Figure 8.**
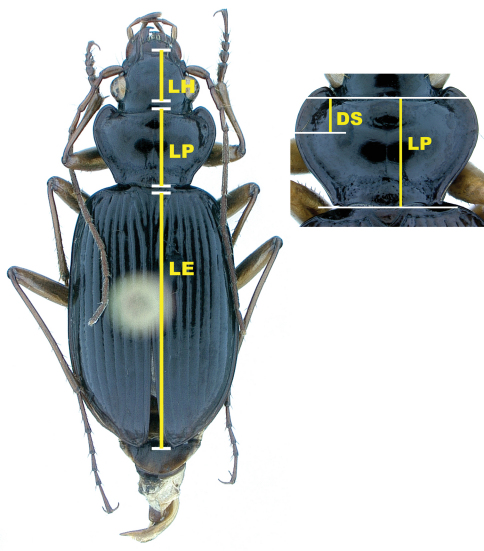
Illustration of measurements; LH = length head; LP = length pronotum; LE = length elytra; DS = longitudinal distance from anterior margin of pronotum to position of left midlateral seta

**Figure 9.**
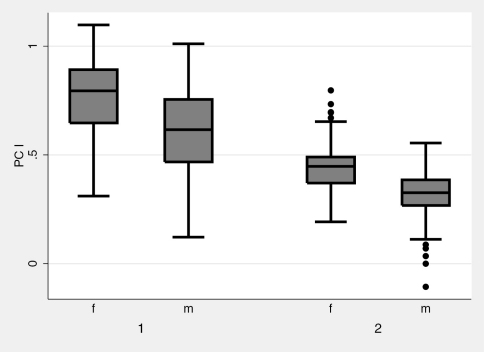
Box plots of body size (measured as the first principal component, PC I) segregated by species and gender. 1 = *Nebria lacustris*, 2 = *Nebria bellorum*. Mid-lines in boxes represent sub-sample distribution median PC I scores (median body size), lower and upper limits of boxes represent 25% and 75% limits of distributions respectively, and upper and lower whiskers represent 5% and 95% limits respectively. *Nebria lacustris* adults are significantly larger than those of *Nebria bellorum*, and females of both species are significantly larger than males.

Based on the observed pattern of geographical variation among *Nebria lacustris* specimens studied, with the only clear phenotypic discontinuity associated with a barrier known to be effective in limiting the ranges of taxa in diverse groups, DHK described the populations south of the French Broad River as a distinct subspecies, *Nebria lacustris bellorum* Kavanaugh (1979), and considered all other populations as included in the nominate subspecies, *Nebria lacustris lacustris* Casey. Bousquet and Larochelle (1993), Ledoux and Roux (2005), and Lorenz (2005) all list *Nebria lacustris* and *Nebria bellorum* as subspecies of a single species and include it in subgenus *Boreonebria* Jeannel (1937), which represents the accepted classification of these taxa from 1979 to the present. However, with the current availability of much more sophisticated computer-based morphometric analytical techniques and straightforward techniques for acquiring and analyzing molecular (comparative DNA sequence) data, it seemed worthwhile to reexamine the the taxonomic status of *Nebria lacustris* using these modern analytical tools. This endeavor seemed all the more appropriate on this occasion because it was begun initially at the prompting of Ross and Joyce Bell.

Through fieldwork by DHK and others (see Acknowledgements) during the period 1994 to 2009, specimens of *Nebria lacustris* were collected at several localities directly into 95% ethanol specifically for comparative molecular analysis. Material collected to date is not sufficient, either in numbers or in geographical coverage, to permit a rigorous phylogeographic analysis for this taxon. However, it was our intent to see if available morphometric and molecular data could be used to address three taxonomic questions. First, are *Nebria lacustris lacustris* and *Nebria lacustris bellorum* distinct enough to warrant status as separate species? Second, is there evidence to suggest that *Nebria lacustris lacustris* is itself a complex of two or more species rather than a single species? Finally, is there morphometric and/or molecular evidence to suggest that the *Nebria lacustris* populations on the shores of Lake Champlain and those in the nearby Green and White Mountains of Vermont and New Hampshire represent distinct evolving units? The purpose of this paper is to report on our findings to date as they pertain to these questions. For convenience and based on additional evidence provided herein, we treat *Nebria lacustris* and *Nebria bellorum* as distinct species throughout this report and refer to this species pair as the *lacustris* species subgroup.

## Materials and methods

**Materials.** This study is based on the examination of more than 2,500 specimens of *Nebria lacustris*, 300 specimens of *Nebria bellorum*, and more than 15,000 specimens of the closely related outgroup taxa used in our molecular analysis, either collected by DHK or borrowed from institutional and private collections (see Kavanaugh 1978 for list of lending individuals and institutions). An additional 12 specimens of *Nebria lacustris*, three specimens of *Nebria bellorum*, and 10 specimens representing eight outgroup taxa were collected, specifically for molecular analysis, by DHK and colleagues (see Acknowledgements). Specimens were collected directly into 95% ethanol, with the ethanol replaced at least twice within a week of capture, and then stored at -20°C (see Table 1 for a list of taxa, specimens, and localities of origin for each). Voucher specimens for the molecular data are deposited in the collection of the Department of Entomology at the California Academy of Sciences.

**Morphological methods**. Morphological features were examined for all specimens acquired using Leitz RS and Leica MZ9.5 stereoscopic dissecting microscopes. Digital photographs of dorsal habitus and pronota were taken using an Auto-montage imaging system by Syncroscopy with a Leica M420 dissecting microscope and a JVC 3-CCD digital camera.

**Morphometric methods**. All measurements were made with a Leitz RS stereoscopic dissecting microscope at a magnification of 16 diameters, using a calibrated ocular grid with a scale interval of 0.1 mm. For each of 1,771 specimens, four distance variables were measured (Fig. 8), including: length of head (LH), measured along midline from apical margin of clypeus to a point opposite posterior margin of the eye; length of pronotum (LP), measured along midline from apical margin to basal margin; longitudinal distance of insertion of left midlateral seta posteriad of midpoint of anterior pronotal margin (DS); and length elytron (LE), measured along midline from apex of scutellum to a point opposite apex of the longer elytron [raw data available from DHK, not included here]. Homogeneity of the pooled samples of both species was examined by principal components analysis (PCA) of these measures. Variables were log-transformed to more closely approximate normal distributions, and PCA was performed on the covariance matrix. PCA specimen scores were used to test size and shape differences between the two species, as well as sexual dimorphism within each species. The relationship between morphology, as described by PCA, and locality data (latitude, longitude, and elevation) was examined for *Nebria lacustris* with individual and multiple regression of PCA scores on those variables. All analyses were performed with Stata 9 for Linux.

**Molecular methods.** DNA was extracted from a single leg from each specimen using the Qiagen DNeasy Blood and Tissue kit and then stored at -20°C. Polymerase Chain Reaction (PCR) was used to amplify regions of the following four nuclear and two mitochondrial genes (with abbreviations used in this paper in parentheses): nuclear 28S ribosomal DNA (28S); nuclear topoisomerase I (TpI); nuclear carbamoylphosphate synthetase region of the “rudimentary” gene (CAD1); nuclear *wingless* (Wg); mitochondrial cytochrome oxidase c subunit I (COI); and a mitochondrial fragment 16S including partial large [16S] subunit rRNA, adjacent tRNA leucine 2, and partial nicotinamide adenine dinucleotide dehydroxygenase subunit 1 [NADH1]. Primers and annealing temperatures used for DNA amplification and sequencing are provided in Table 2.

**Table 2.** Primers and annealing temperatures used for DNA amplification (PCR) and sequencing

Gene	Primer	Sequence (5’ to 3’)	PCR annealing temperature	Source
28S	LS58F	5-GGGAGGAAAAGAAACTAAC-3	54°C	Ober 2002
	LS998R	5-GCATAGTTCACCATCTTTC-3	54°C	Ober 2002
TpI	TP643F	5-GACGATTGGAARTCNAARGARATG-3	58°C	Wild and Maddison 2008
	TP675F	5-GAGGACCAAGCNGAYACNGTDGGTTGTTG-3	60°C	Wild and Maddison 2008
	TP932R	5-GGWCCDGCATCDATDGCCCA-3	58°C/60°C	Wild and Maddison 2008
CAD1	CAD1F	5-GARCAYACAGCNGGNCCNCAAGA-3	57°C/52°C/45°C	W. Moore unpublished
	CAD1R	5-AANGGRTCNACRTTTTCCATATT-3	57°C/52°C/45°C	W. Moore unpublished
Wg	Wg550F	5-ATGCGTCAGGARTGYAARTGYCAYGGYATGTC-3	52°C	Wild and Maddison 2008
	Wg578F	5-TGCACNGTGAARACYTGCTGGATG-3	52°C	Wild and Maddison 2008
	WgAbRZ	5-CACTTNACYTCRCARCACCARTG-3	52°C	Wild and Maddison 2008
	WgAbR	5-YTCGCAGCACCARTGGAA-3	52°C	modified from Wild and Maddison 2008
COI	Jer	5-CAACATTTATTTTGATTTTTTGG-3	51°C	Simon 1994
	Pat	5-TCCAATGCACTAATCTGCCATATTA-3	51°C	Simon 1994
16S	16SAR	5-CGCCTGTTTAACAAAAACAT-3	56°C	Papadopoulou 2009
	ND1A	5-GGTCCCTTACGAATTTGAATATATCCT-3	56°C	Papadopoulou 2009

Nested and hemi-nested PCR strategies were used for Wg and TpI, respectively. Initial PCRs for Wg and TpI were carried out using the primer pairs Wg550F/WgAbRZ and TP643F/TP932R, respectively. Internal PCR products were then produced using the initial PCR product as template and the primer pairs Wg578F/WgAbR and TP675F/Wg932R. The cycling conditions for CAD1 involved a three-cycle, three-step program using the annealing temperatures 57°C, 52°C and 45°C, to increase specificity for the desired product. For detailed PCR conditions, please contact the authors. Cycle sequencing was performed using BigDye v3.1 chemistry and run on an Applied Biosystems 3130xl Genetic Analyzer. Assembly of chromatograms (contigs) for each sequence and initial base calls were made using Sequencher v4.9 (Gene Codes Corporation, Inc.). Multiple peaks or different peaks at a single position in complimentary fragments were coded using IUPAC/GCG ambiguity codes. Sequences have been deposited in GenBank with accession numbers JN847505 through JN847654.

Due to the low levels of observed genetic variation, sequence alignment of the protein coding genes TpI, CAD1, Wg and COI was performed by eye with minor manual adjustments using MacClade v4.08 (Maddison and Maddison 2005). Alignment of protein coding genes was further corroborated by translating sequences into amino acids in MacClade v4.08 (Maddison and Maddison 2000) and checking for stop codons. Alignment of the ribosomal genes 28S and 16S was performed using MAFFT v6.818 (Katoh et al. 2009) with default parameters. Best fit models of nucleotide evolution were selected for each gene and codon position (see Table 3) using the Akaike Information Criterion (Akaike 1973) in MrModeltest v2.2 (Nylander 2004).

**Table 3.** Evolutionary models used for Bayesian analysis as selected by the Akaike Information Criterion in MrModeltest v2.2 (Nylander 2004).

Gene	Codon	Model
28S		SYM+I
TPI	Position 1	HKY+I
TPI	Position 2	HKY
TPI	Position 3	K80+Γ
CAD1	Position 1	GTR
CAD1	Position 2	HKY+I
CAD1	Position 3	GTR+Γ
Wg	Position 1	F81
Wg	Position 2	F81
Wg	Position 3	HKY+I
COI	Position 1	GTR+I
COI	Position 2	HKY
COI	Position 3	GTR+Γ
16S		GTR+I+Γ

**Phylogenetic inference from molecular data**. Maximum likelihood, Bayesian, and parsimony analyses were conducted on separate matrices of data for each of the six genes and on a matrix of concatenated data for all genes. All matrices consisted of data for 25 specimens, including ten representing outgroups and fifteen representing the *lacustris* species subgroup (i.e., *Nebria lacustris* and *Nebria bellorum*) (see Table 1). Outgroup selection was based on preliminary results from a phylogenetic analysis [in progress, DHK] of morphological and molecular data for more than 170 taxa representing the supertribe Nebriitae. *Nebria gouleti* Kavanaugh, *Nebria hudsonica* LeConte and *Nebria lacustris* comprise the *hudsonica* species group of subgenus *Boreonebria* Jeannel. *Nebria nivalis* (Paykull), *Nebria baicalica* Motschulsky, *Nebria subdilatata* Motschulsky, *Nebria gyllenhali* Schönherr, and *Nebria crassicornis* Van Dyke represent closest outgroups within subgenus *Boreonebria*, and *Nebria brevicollis* (Fabricius) represents subgenus *Nebria* sensu stricto, which is the sister group to *Boreonebria* in most of the gene trees examined to date.

Maximum likelihood analysis was performed using the Randomized Accelerated Maximum Likelihood algorithm in RAxML v7.0.4 (Stamatakis 2006). Partitioned analyses in RAxML were limited to the general time-reversible model with gamma distributed among site rate variation (GTR + Γ). One thousand non-parametric bootstrap replicates were conducted for each search, using the rapid bootstrap algorithm as employed in the program. Both independent gene trees and a concatenated data set were analyzed, with protein coding genes fully partitioned by codon.

Bayesian analysis was performed using MrBayes v. 3.1.2 (Huelsenbeck and Ronquist 2001) at the California Academy of Sciences Center for Comparative Genomics (CCG) lab (see Table 3 for best fit models used for each gene and codon positions). Analysis proceeded using 4 independent runs until the standard deviation of split frequencies fell below 0.01. Stationarity was evaluated by examining the stability of posterior probabilities for nodes of each MCMC run using the Cumulative and Compare plots in “Are We There Yet?” (http://ceb.csit.fsu.edu/awty; Wilgenbusch et al. 2004). The distributions of each parameter for all runs were also visually inspected in Tracer v.1.5 to insure that they were well-sampled and effective sample sizes (ESS) were above 200. The first 25% of trees were discarded from the posterior distributions of each analysis after examination using the Cumulative plot in AWTY.

Parsimony analyses were performed in PAUP* (Swofford 2003) using 1000 iterations of a heuristic search, with random taxon addition and tree bisection-reconnection (TBR) branch-swapping. Nonparametric bootstrap support values were calculated using 1000 replicate searches with random taxon addition.

**Chorological methods.** Geographical coordinates were recorded for localities for all2,500 specimens examined, including the 1,771 specimens from which measurements were taken. For the majority of specimens, coordinates were not included among original label data, so these had to be estimated retrospectively. This was accomplished, using Google Earth, by first locating each locality as precisely as permitted by the label data, including elevation if recorded, and then locating appropriate habitat for the species (namely rivers and streams with evident rocky or graveled banks) at that locality. For specimens for which label data did not include elevation, this variable was estimated from values provided by GoogleEarth for the collections sites estimated by label locality data and habitat only. Ultimately, the combination of label locality, elevation, and habitat was sufficient for a good estimate of the actual site of collection for virtually all specimens.

## Results

**Morphological evidence.** More than any other *Nebria* taxon in North America, members of the *lacustris* species subgroup exhibit a complex pattern of individual, interpopulational, and geographical variation in adult morphology. This variation includes conspicuous differences in overall body size and relative size and shape of the pronotum and, to a lesser extent, elytral shape, convexity of elytral intervals, and elytral microsculpture. As is typical of species of subgenus *Boreonebria*, the subgenus of which this taxonis a member, there is little or no apparent variation in male or female genitalic morphology associated with this variation in external features. The pattern of variation in size is discussed below under Morphometric evidence, but we address variation in the other features here.

Even very slight differences in pronotal shape and/or relative size can impart a distinctly different gestalt to beetles that are otherwise quite similar. Such is particularly true for adults of the *lacustris* species subgroup. Perhaps the most conspicuous single varying pronotal feature is depth of the basolateral sinuations of the lateral margins. They are absent or very shallow in specimens from Bayfield Wisconsin, for example (Fig. 1), but evident to a greater, but varied, extent in specimens from most other areas (Fig. 2 and  4–7). Their depth varies within populations, as does their length. However, specimens from lowland areas just west of the Appalachian Mountains chain (e.g., Fig. 4 and 7) and from the southern Appalachian highlands (Fig. 6) have shorter and deeper sinuations than most specimens from all other areas. Most specimens from the mountains of Vermont and New Hampshire and from localities farther north to the north shore of the Saint Lawrence estuary in Québec have these sinuations distinctly long and shallow. Another conspicuous difference among specimens is in the shape and convexity of the lateral margins at the widest part of the pronotum. In specimens from lowland areas just west of the Appalachian chain (Fig. 4 and 7), for example, these regions of the margins are broadly and evenly rounded, whereas in most specimens from other areas, they are more rounded anteriorly and less rounded posterior to the widest point (at the insertion of the midlateral pronotal seta) (e.g., Fig. 5 and 6). In still other individuals, the margins are less rounded both anterior and posterior to the widest point (e.g., Fig. 2). Another conspicuous difference is in the relative length of the pronotum. Specimens from the southern Appalachian highlands (Fig. 6) have a very short, broad pronotum compared with most specimens from all other areas, and particularly those from the northeasternmost part of the range. Otherwise most similar in size and shape to southern Appalachian specimens, specimens from the northeast (Fig. 5) have pronota that are particularly long and narrow. Subtle individual and interpopulational differences are also apparent in the length, width, and shape of the anterior pronotal angles, the shape of the posterior pronotal angles, and in the width of the lateral explanation of the pronotum, but we have found no clear pattern to this variation.

*Nebria bellorum* specimens, from the southern Appalachian highlands, have an elytral silhouette that is subrectangular, with lateral margins relatively straight and subparallel (Fig. 6). Almost all specimens from all other areas, especially those from adjacent areas to the north and west, have more distinctly rounded lateral margins and, therefore, a more rounded elytral silhouette (Fig. 1–2, 4, 5, and 7). The elytral intervals are slightly less convex in *Nebria bellorum* specimens than in most specimens from all other areas. Although the elytral microsculpture for all *lacustris* species subgroup members is comprised of markedly transverse meshes, *Nebria bellorum* specimens have the meshes more deeply impressed than specimens from most other areas. This feature imparts a slightly duller luster to the elytra of *Nebria bellorum* adults than that of specimens from other areas, which are shinier with slight iridescence.

**Morphometric evidence.** Principal components analysis (PCA) of the pooled species and locality samples yielded two components that account for 97.66% of variance in the data (Table 4). The first principal component, PC I (85.48% of variance), may be interpreted as a measure of allometric size because of the positive loadings of all the morphometric variables. Allometry is indicated by the greater magnitude of the DS score, suggesting that this variable contributes more significantly to overall increase in body size relative to the other variables. The second component, PC II (12.19% of variance) is a shape variable, contrasting the relative magnitudes of the DS measure versus the other three distance measures.

**Table 4.** Principal components analyses of morphometric variables for *Nebria lacustris* and *Nebria bellorum*. Variables: LH = length head; LP = length pronotum; LE = length elytra; DS = distance left midlateral seta from anterior margin. All variables were log-transformed and covariance matrices analyzed. First analysis, *Nebria lacustris* and *Nebria bellorum*: PC I – 85.48% and PC II – 12.19% of variance respectively. Second analysis, *Nebria lacustris* only: PC I – 86.15% and PC II – 11.07% of variance respectively.

	*Nebria lacustris* and *Nebria bellorum*		*Nebria lacustris* only	
Variable	PC I	PC II	PC I	PC II
LH	0.356	0.473	0.306	0.521
LP	0.356	0.412	0.297	0.417
LE	0.346	0.485	0.291	0.538
SD	0.792	-0.609	0.856	-0.514

**Figure 10.**
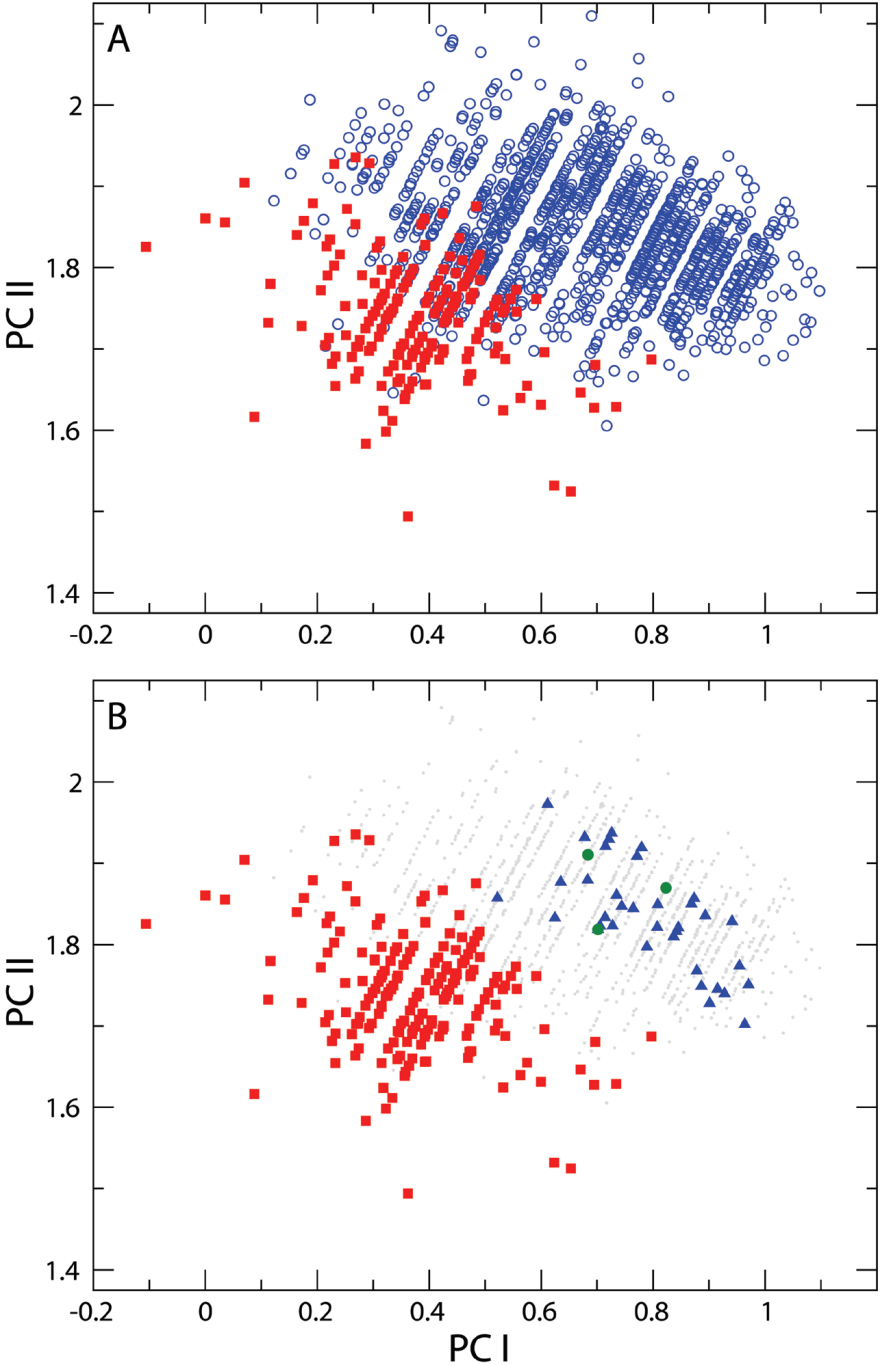
**A** Principal components analysis of distance measurements on *Nebria lacustris* (blue open circles) and *Nebria bellorum* (filled red squares). PC I (x axis) is a measure of allometric size, while PC II is a shape contrast between the DS measure and all others **B** Upper figure repeated to highlight dissimilarity between geographically proximate specimens of *Nebria bellorum* and specimens of *Nebria lacustris* from North Carolina, Tennessee, Kentucky, and Virginia (filled blue triangles). Geographically distant specimens from Iowa are indicated by filled green circles, and fall within the central area of *Nebria lacustris*’s morphometric distribution (see text for further discussion). All other specimens of *Nebria lacustris* are shown as grey dots.

Females are significantly larger than males in both species (for *Nebria lacustris*, n=1558, t=18.207, p<0.0001; for *Nebria bellorum*, n=213, t=8.3483, p<0.0001) (Fig. 9), and both females and males of *Nebria lacustris* are significantly larger than individuals of *Nebria bellorum* (nested ANOVA, F=348.57, p<0.0001). The species also differ significantly in shape (n=1771, t=19.8766, p<0.0001), that is, PC II (Fig. 10).The higher scores of specimens assigned to *Nebria lacustris* reflect the relatively smaller value of DS, or relatively greater value of the other three variables, in this species compared to *Nebria bellorum*. The strong morphometric discrimination indicates that this is a useful distinguishing character between the species.

**Figure 11.**
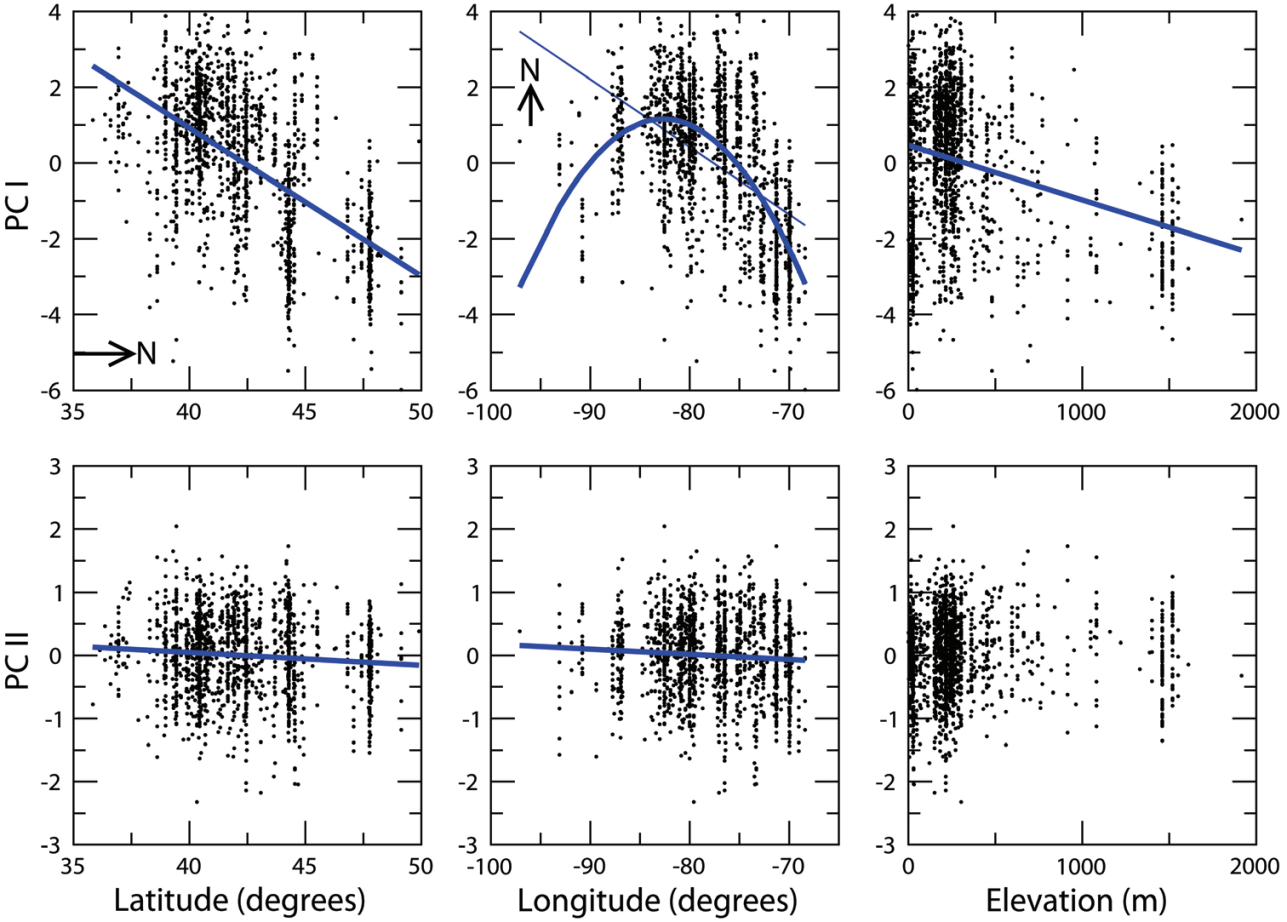
Principal components analysis scores of *Nebria lacustris* versus location and elevation. Body size (PC I) varies significantly with the three factors, while the independent shape variable, PC II, varies significantly with location. Individual regression lines drawn on plots represent individual relationships within multiple regression models (see text for explanation). A quadratic model is selected for the relationship between PC I and longitude because a first order linear model results in non-normally distributed residuals (linear model is shown as light blue straight line). “N” on plots indicates cardinal direction north.

Testing any hypothesis relating morphological variation of *Nebria lacustris* to locality requires the simultaneous consideration of latitude, longitude and elevation, because of potential covarying effects of those variables. Nevertheless, the manner in which the morphological variables (PC I and PC II) covary separately with each variable is a useful first guide prior to performing a multiple regression. In this case, a second PCA was conducted of *Nebria lacustris* only to remove any confounding effects of *Nebria bellorum* on the covariance matrix. The second analysis preserves the findings of the first analysis, in that PCI is an axis of allometric size and PC II is a shape variable similar to PC II in the first analysis (Table 4). Figure 11 illustrates the relationship between PC I and PC II specimen scores, and latitude, longitude and elevation. Size (PC I) decreases with increasing latitude and elevation, but has a more complicated relationship with longitude. Overall, size increases with longitude, reaching a maximum at approximately 80° W, declining thereafter. The entire set of relationships was therefore measured with a multiple regression, including a quadratic term for longitude, and the three locality variables account for 46% of size variation in *Nebria lacustris* (R-squared=0.46, F=330.54, p<0.0001). There is a less strong but significant relationship of the shape variable (PC II) to locality (R-squared=0.0362, F=19.42, p<0.0001), though the relationship is to latitude and longitude only, with no change in shape with elevation. PC II scores decrease with increasing latitude, indicating a relative increase in the magnitude of the DS measure with increasing latitude. It must be emphasized, however, that location explains only 3.62% of the shape variance measured.

**Molecular evidence.** Molecular data generated from PCR, sequencing, and alignment of trimmed fragments recovered for each of the six genes sampled are summarized in Table 5. Perhaps the most surprising finding was the overall low genetic divergence observed among the samples, including both those representing the *lacustris* species subgroup and those representing outgroup taxa, particularly the other species of the *hudsonica* species group, *Nebria gouleti* and *Nebria hudsonica.* For example, not a single nucleotide difference was found among the 948 bp trimmed fragments for 28S for all *hudsonica* group representatives. The combined trimmed fragment lengths for the six genes for *lacustris* species subgroup representatives totaled 4605 bp; but, of these, a total of only 18 (0.39%) showed any base differences among the samples for this taxon. The highest relative diversity within the *lacustris* species subgroup was found in Wg, in which only five of 453 sites (1.10%) showing nucleotide divergence.

**Table 5.** Summary of molecular results. Number of sites in the aligned matrices and number of base pairs in *Nebria lacustris* species subgroup sequence fragments represent trimmed fragments.

	28S	TpI	CAD1	Wg	COI	16S	totals
total sites in aligned matrix	1034	755	802	453	822	829	4695
bases in *lacustris* species subgroup sequence fragments	948	755	802	453	822	825	4605
sites with base differences within the *lacustris* species subgroup	0	3	2	5	6	2	18
% sites with base differences within the *lacustris* species subgroup	0	0.40	0.25	1.10	0.73	0.24	0.39
unique amino acid differences for *hudsonica* group	-	0	1	0	1	-	2
unique base differences for *hudsonica* group	3	1	9	1	4	0	18
unique deletions for *hudsonica* group	6	0	0	0	0	1	7
unique amino acid differences for the *lacustris* species subgroup	-	0	0	0	0	-	0
unique base differences for the *lacustris* species subgroup	0	0	2	0	3	5	10
unique insertions for the *lacustris* species subgroup	0	0	0	0	0	1	1
unique base differences for *Nebria bellorum*	0	0	0	0	0	1	1
unique base differences for *Nebria lacustris* from Iowa	0	1	0	0	0	1	2
unique base differences between Lk. Champlain and Green Mts. samples	0	0	0	0	0	0	0

**Figure 12.**
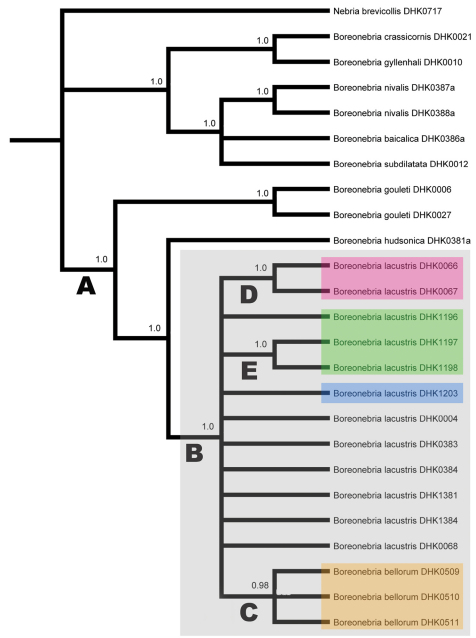
Majority-rule consensus tree from fully-partitioned Bayesian analysis (see Table 3 for evolutionary models used) of concatenated data set; only branches supported by posterior probability values of 0.95 or greater (as noted on branches) indicated. Capital letters (A through E) denote branches discussed in the text. Samples within colored boxes designate the following: gray = specimens of the *lacustris* species subgroup; red = specimens from Iowa; green = specimens from the Green Mountains, Vermont; blue = specimen from the shore of Lake Champlain, Vermont; orange = specimens from the southern Appalachian highlands (*Nebria bellorum*).

Phylogenetic analyses using parsimony, maximum likelihood, and Bayesian methods produced very similar results, so we present here only the majority-rule consensus tree from the fully-partitioned Bayesian analysis of the concatenated dataset (Fig. 12). Only branches supported by Bayesian posterior probability values (bpp) of 0.95 or more are noted.

The analysis recovered a monophyletic *hudsonica* species group (bpp = 1.0) (branch “A” in Fig. 12), members of which shared 18 unique bases, one unique deletion (in 28S), and two unique amino acid differences (one each in the CAD1 and COI sequences) relative to their outgroup. Relationships among *Nebria gouleti*, *Nebria hudsonica*, and the *lacustris* species subgroup are not as clear as suggested by the Bayesian tree for concatenated data. Only the trees for concatenated data and CAD1 returned *Nebria hudsonica* as the sister group to the *lacustris* species subgroup and *Nebria gouleti* as the sister to that pair. The strict consensus parsimony tree for COI returned *Nebria gouleti* and the *lacustris* species subgroup as sister species, with *Nebria hudsonica* as their sister group, and relationships among these taxa remained unresolved in all trees for 28S, TpI, Wg, and 16S and the likelihood and Bayesian trees for COI.

The monophyly of the *lacustris* species subgroup (branch “B” in Fig. 12) is also strongly supported (bpp = 1.0). Taxon members in our sample share ten unique bases and one unique insertion (in 16S), but no unique amino acid differences. However, evidence of fixed genetic divergence within this taxon is limited to only two instances. First, specimens from the southern Appalachian highlands (branch “C” and highlighted in the orange box in Fig. 12) form a monophyletic group in our sample (bpp = 0.98) and share a single unique base in their 16S sequences. We have identified this monophyletic group as *Nebria bellorum*. Second, the two specimens from Iowa in our sample (branch “D” and highlighted in the red box in Fig. 12) also form a monophyletic group (bpp = 1.0) and share two unique bases, one in each of their TpI and 16S sequences.

We found four additional instances of genetic divergence within the *lacustris* species subgroup. (1) Two of three specimens in our sample from Ridley Brook, Vermont (DHK1197 and DHK1198) (branch “E” in Fig. 12), share a unique 1^st^ position base in their Wg sequences, which corresponds to a shared unique amino acid difference from all other samples. The remaining specimen from that locality (DHK1196) has the same base at this site as all other remaining *hudsonica* group samples. (2) One specimen (DHK0004) out of the five in our sample from the Plummers Island, Maryland has a 1^st^ position base and resulting amino acid coded for in its COI sequence that is not shared with any other *Nebria lacustris* specimens but is, instead, shared with the specimen of the most distance outgroup in the analysis, *Nebria brevicollis* (DHK0717). (3) Another of the five Plummers Island specimens (DHK0384) has a unique 2^nd^ position base in its COI sequence that codes for an amino acid not shared with any other specimen in our sample. (4) The single specimen from Linville River, North Carolina (DHK0068) has a unique 2^nd^ position base in its Wg sequence that codes for an amino acid not shared with any other samples. These four instances, however, provide no evidence of additional fixed differences (divergence) within *Nebria lacustris*, including between our sample from the Vermont shore of Lake Champlain (highlighted in blue in Fig. 12) and all the specimens in the sample from Ridley Brook in the adjacent Green Mountains (highlighted in green in Fig. 12).

**Distributional evidence.** As noted in the Introduction, *Nebria lacustris* sensu lato occupies a broad geographical range in eastern North America (Fig. 3). It also occupies a broad range of elevations (from at or near sea level at Angelsea, New Jersey, to 1610 m on Mount Washington, New Hampshire) as well as habitats. These beetles are found on lakeshores and on the banks of streams of all sizes, where there are accumulations of loose rocks under which these nocturnal predators can hide during daylight hours. In these habitats, they are usually found only where their shelters are shaded from direct sun by overhanging banks or trees.

In the northeastern and central parts of the range of this taxon, locality records are many and the distribution of localities appears to be (or at least have been prior to European settlement) virtually continuous in rocky riparian and lacustrine habitats. The ranges of the Appalachian Mountains system appear to have presented no barrier to the East-West distribution of this taxon at latitudes at or north of 37°N. By contrast, in the westernmost part of the range, recorded populations are few and widely separated. Because the region has been extensively sampled by entomologists for more than a century, this dearth of known localities probably represents a true paucity of sites with suitable habitat (namely, rocky, as opposed to sandy or fine gravel, stream banks).

**Figure 13.**
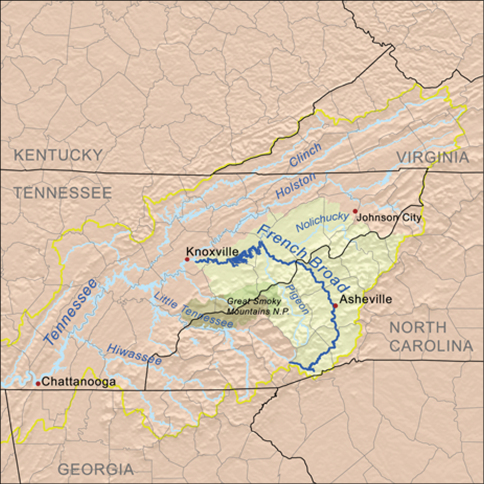
Map showing the location and extent of the French Broad River in North Carolina and Tennessee. Map copyright Karl Musser, distributed under a Creative Commons Attribution-Share Alike 2.5 Generic license [http://creativecommons.org/licenses/by-sa/2.5/deed.en] and available at http://en.wikipedia.org/wiki/File:Frenchbroadrivermap.png

In the southernmost part of the range, populations are restricted to the Appalachian highlands, generally at elevations well above 500 m. It is in this area that the only apparent significant barrier to continuity of distribution is found—namely, the French Broad River. This river has its headwaters on the southeastern slope of Pisgah Ridge and eastern slope of Tanasee Ridge in Transylvania County, North Carolina, and flows for more than 300 km, first east, then northwest, then west into Tennessee, where it joins the Tennessee River as one of the latter’s main tributaries (Fig. 13). Thus, the river’s course cuts completely across the main axis of the Appalachian chain, serving as a broad, warm, low-elevation (ca. 500-600 m) gap in the mountain chain and as a barrier between beetle populations restricted to cooler, higher elevations and shaded habitats on opposite sides of this gap. All known populations of *Nebria bellorum* occur south of this gap, isolated from all known populations of *Nebria lacustris* north of it.

## Discussion

Morphological, morphometric, molecular, and chorological evidence each contributes information useful in answering the original questions of our study. However, there is need for additional research along each of these lines of evidence in the future to better understand *Nebria lacustris*, the patterns of its morphological and genetic diversity, and also its evolutionary history. Although not among the original questions we posed, it was encouraging to see that the monophyly of both the *hudsonica* species group and of *Nebria lacustris* sensu lato is strongly supported by our molecular data. But what about those original questions? Our answers, provided in detail below, are summarized in Table 6.

**Table 6.** Summary of evidence. Parentheses denote support from only one or two of the three (Bayesian, maximum likelihood, and parsimony) methods of phylogenetic analysis applied.

Question	morphological evidence	morphometric evidence	chorological evidence				molecular evidence			
				28S	TpI	CAD1	Wg	COI	16S	Concat
Monophyly of *hudsonica* sp. group?	-	-	-	yes	no	yes	(yes)	yes	(yes)	yes
Monophyly of the *lacustris* species subgroup?	-	-	-	no	(yes)	yes	no	yes	yes	yes
Distinctiveness of *Nebria bellorum*?	yes	yes	yes	no	no	no	no	no	yes	yes
Distictiveness of Iowa samples of *Nebria lacustris*?	no	no	no	no	no	no	no	no	yes	(yes)
Distinctiveness of *Nebria lacustris* from Lake Champlain versus adjacent mountains?	no	no	no	no	no	no	no	no	no	no

**Are *Nebria lacustris* sensu stricto and *Nebria bellorum* sufficiently distinct to warrant status as separate species?** Better imaging equipment than was available in the early 1970’s has provided an increased ability to both readily compare large numbers of specimens and better visualize character state definitions. Consequently, *Nebria bellorum* and *Nebria lacustris* are more easily and widely distinguished on morphological grounds than before. Most significantly, application of more sophisticated morphometric analysis (PCA) to the dataset gathered during the 1970’s clearly demonstrates that *Nebria bellorum* and *Nebria lacustris* are morphometrically distinct based on the mensural parameters analyzed. We suspect that morphometric analyses of additional pronotal shape features will provide even stronger evidence of the distinctiveness of these two taxa, but the appropriate measurements have not yet been taken. The molecular evidence pertinent to this question is not as conclusive as we had hoped for or expected. The single unique base shared by the three *Nebria bellorum* specimens in our sample suggests a fixed difference between members of this taxon and those of all other *Nebria lacustris* populations, but this should be tested thoroughly with additional *Nebria bellorum* specimens, with additional specimens of *Nebria lacustris* from populations closest to the range of *Nebria bellorum*, and with additional fast-evolving genes. Finally, the examination of more than 500 additional specimens that were not available during the 1970’s has also helped to better define the pattern of geographical variation in the *lacustris* species subgroup and also supports the previously observed geographical discontinuity (vicariance) between *Nebria bellorum* and *Nebria lacustris* across the French Broad River valley and the endemism of *Nebria bellorum* in the southern Appalachian highlands.

Based on consideration of all the lines of evidence outlined above, we suggest that *Nebria lacustris* and *Nebria bellorum* warrant status as distinct species, which represents a NEW STATUS for the latter taxon. Analysis of the available molecular evidence results in an unresolved polytomy; and, depending upon how that polytomy is resolved, treating *Nebria bellorum* as a distinct species may render *Nebria lacustris* as either a monophyletic or a paraphyletic species. A paraphyletic *Nebria lacustris* might be expected if, as we suspect, *Nebria bellorum* represents a relatively recent product of peripheral isolation and subsequent divergence from the ancestral *lacustris* stock and complete lineage sorting has not yet occurred (Funk and Omland 2003). So little nucleotide divergence was found throughout *Nebria lacustris* sensu lato in our dataset that the single unique 16S base difference appears all the more significant. Clearly, *Nebria bellorum* is a taxon distinctly defined on morphological, morphometric, and chorological grounds and represents an independently evolving taxon. We have no solid evidence at present to suggest that *Nebria lacustris* is not also a single, independently evolving taxon (but see below).

**Is there evidence that *Nebria lacustris* sensu stricto is itself a complex of two or more species rather than a single species?** Both morphological and morphometric evidence shows marked variation in size, overall shape, aspects of pronotal shape, and other features in *Nebria lacustris* over its geographical range. Some of the variation is seen within populations, but much of it has a complex geographical component. The morphometric evidence is particular helpful in better defining the pattern of geographical variation, which clearly shows that size (PC I) decreases with elevation and latitude (Fig. 11). The same latitudinal pattern appears, but less distinctly, with respect to the pronotal shape character (PC II). The longitudinal component seems to be more complex than the other two, but this may be due, at least in part, to the fact that at the extreme eastern and western limits of the range, localities where the species occurs are in increasingly more northern areas (Fig. 3). The extent to which ecophenotypic variation, rather than genetic variation, contributes to this pattern is unknown. As noted in the morphometric results above, location appears to account for only a small portion (only 3.62%) of the shape variance measured, so there must be a greater genetic component involved in determining shape than we have been able to see in the six genes sampled. Although the known localities for *Nebria lacustris* in the western part of its range are more widely scattered, no particular geographic or physiographic features stand out as evident barriers to distribution within the overall range. Hence, there do not appear to be any clearly defined subunits within *Nebria lacustris* based on morphological, morphometric, or geographical grounds.

Our evidence for genetic diversity in *Nebria lacustris* over its range is admittedly meager and demonstrates surprisingly low divergence overall, with only 0.37% of sites varying over the concatenated dataset. Most of the nucleotide and amino acid differences were confined to single genes in single individuals and in only one instance represented all specimens from a single locality. That exception involves the two specimens from the Iowa River in eastern Iowa, which, as noted in our results, share unique single base differences in TpI and 16S sequences. Clearly, additional specimens from northeastern Iowa are needed to determine if this really represents a fixed difference locally. We also had no other specimens available from west of North Carolina and Vermont, so sequence data for these genes is completely lacking for populations in the intervening region. Nonetheless, the discovery of unique genetic traits, in both nuclear and mitochondrial genes, in this area suggests an interesting possibility. The Iowa River locality is part of a region widely known as the “Driftless Zone”—an area of more than 40,000 km^2^ covering the extreme southeastern corner of Minnesota, the southwestern half of Wisconsin, northeastern Iowa, and extreme northwestern Illinois and centered along the Mississippi River in this region. Based on the absence of glacial deposits attributable to late glacial advances, as well as a physiography atypical of recently glaciated areas, it has been widely proposed and accepted that the area itself remained ice-free while more or less surrounded by lobes of the continental ice sheet throughout at least the last major glaciation and perhaps earlier glacial advances as well (Frye and Willman 1973). The Vernon County [Wisconsin] Land and Water Conservation Department’s website (http://www.co.vernon.wi.gov/LWCD/driftlessArea.htm) describes present day habitat in the area as follows: “In addition to the curious topography of steep slopes and cliffs, there are unique habitats, the algific (cold air) talus (loose rock) slopes. These slopes remain cool throughout the year and are home to rare species of plants and animals”. The presence of such unique habitats may explain the occurrence of *Nebria lacustris* today so far west and in a region where no other *Nebria* species are known or suspected to occur. However, extant populations in this region also may have descended from a glacial population that survived at least the last glacial advance in a refugium in the Driftless Zone. A detailed phylogeographic study of *Nebria lacustris* throughout its range, but especially in this and adjacent regions, might provide evidence with which to test this hypothesis.

**Is there evidence to suggest that the *Nebria lacustris* populations on the shores of Lake Champlain and those in the nearby Green and White Mountains of Vermont and New Hampshire represent distinct evolving units?** Based on our answer to the second question, the answer is no. This does not refute the fact that many specimens from Lake Champlain are distinctly larger and look very different from specimens along the small, shaded brooks and streams at higher elevations in the adjacent Green and White Mountains. While the size differences are consistent with the overall pattern shown by the morphometric analysis (for PC I, Fig. 11), the shape differences are more difficult to explain and interpret. It may be that the steep physiographic gradient between Lake Champlain and the adjacent Green Mountains limits direct genetic exchange between populations in these two areas, but that the physiographic gradient is circumvented by gene exchange north and south of the steepest part of the gradient. Our meager molecular evidence suggests no genetic discontinuity, but this should be tested further with additional sampling in many lowland and upland localities throughout the region.

**Directions for Future Research**. As noted repeatedly in our discussion above, we suggest that a comprehensive phylogeographic study of the *lacustris* species subgroup would prove most useful for gaining a understanding of the basis of the present morphological and genetic diversity of this species, as well as its evolutionary and biogeographic history. Such a study would best be carried out by someone residing in or near the range of this taxon so that the extensive field work and sampling required to support such a study might be undertaken efficiently and inexpensively. Specimens collected for use in the molecular analyses could also be used in an expanded morphometric analysis, particularly for several additional features related to pronotal shape. Results from such molecular and morphometric analyses would complement one another and advance our understanding of this species and its history dramatically; and the lead author is eager to assist anyone seriously interested in such a project.
